# Epigenetic Targets in Synovial Sarcoma: A Mini-Review

**DOI:** 10.3389/fonc.2019.01078

**Published:** 2019-10-18

**Authors:** Ryland Hale, Sami Sandakly, Janet Shipley, Zoë Walters

**Affiliations:** ^1^Translational Epigenomics Team, Human Development and Health, Faculty of Medicine, University of Southampton, Southampton, United Kingdom; ^2^Sarcoma Molecular Pathology Team, Divisions of Molecular Pathology and Cancer Therapeutics, The Institute of Cancer Research, London, United Kingdom

**Keywords:** synovial sarcoma, SS18-SSX, epigenetics, therapeutic targets, SWI/SNF, PRC1, PRC2, chromatin remodeling

## Abstract

Synovial Sarcomas (SS) are a type of Soft Tissue Sarcoma (STS) and represent 8–10% of all STS cases. Although SS can arise at any age, it typically affects younger individuals aged 15–35 and is therefore part of both pediatric and adult clinical practices. SS occurs primarily in the limbs, often near joints, but can present anywhere. It is characterized by the recurrent pathognomonic chromosomal translocation t(X;18)(p11.2;q11.2) that most frequently fuses SSX1 or SSX2 genes with SS18. This leads to the expression of the SS18-SSX fusion protein, which causes disturbances in several interacting multiprotein complexes such as the SWItch/Sucrose Non-Fermentable (SWI/SNF) complex, also known as the BAF complex and the Polycomb Repressive Complex 1 and 2 (PRC1 and PRC2). Furthermore, this promotes widespread epigenetic rewiring, leading to aberrant gene expression that drives the pathogenesis of SS. Good prognoses are characterized predominantly by small tumor size and young patient age. Whereas, high tumor grade and an increased genomic complexity of the tumor constitute poor prognostic factors. The current therapeutic strategy relies on chemotherapy and radiotherapy, the latter of which can lead to chronic side effects for pediatric patients. We will focus on the known roles of SWI/SNF, PRC1, and PRC2 as the main effectors of the SS18-SSX-mediated genome modifications and we present existing biological rationale for potential therapeutic targets and treatment strategies.

## Introduction

Synovial sarcoma (SS) is a rare (1–3 cases per 1,000,000) aggressive high-grade malignancy mainly observed in adolescents and young adults, with a third of all SS cases occurring in patients under the age of 20. Pediatric SS predominantly occurs in the limbs (1), but can occur anywhere. This disease is characterized by the pathognomonic reciprocal t(X;18)(p11.2;q11.2) chromosome translocation, which leads to the fusion of the SS18 (formerly SYT) gene to one of three SSX genes (2–6). In two thirds of cases SS18 is fused to SSX1, with SSX2 as a fusion partner in most other cases, whilst a fusion with SSX4 occurs rarely. SS18 contains an SNH domain and a QPGY domain, both of which are present in the fusion protein. The SSX proteins contain a Krüppel associated box (KRAB) repression domain at their N-terminus that is excluded from the 79 C-terminal amino-acids (a.a.) fusing to SS18 (2–4, 7). SS18 functions as a co-activator of transcription, however the SSX proteins have been shown to moderate repression, allowing the oncoprotein the ability to activate and repress gene expression.

Other than the translocation, SS tumors are mutationally quiet. This is especially true in the pediatric setting (8). Furthermore, pediatric SS is associated with a better prognosis compared to its adult counterpart (9–16). Despite this, both patient populations face the same poor prognosis in metastatic context, which is associated with increased tumor genomic instability (8). A difference is seen in the rate of patients with metastases at diagnosis, reflected by 5–11% of pediatric patients (1, 17) compared to the 50% observed in newly diagnosed adult patients (1, 17, 18).

SS can be classified into three distinct histological subtypes (19). Monophasic SS is characterized by spindle cells of mesenchymal differentiation. Biphasic SS shows evidence of both epithelial and mesenchymal differentiation, resulting in epithelial-like structures among the spindle cells. The third subtype, undifferentiated SS, is characterized by a lack of differentiation. Currently, there is no clear evidence for a link between SSX fusion partner and histological subtype.

Like most other soft tissue sarcomas, systemic therapy for pediatric SS relies on heavy use of doxorubicin and ifosfamide associated with radiotherapy for local control (20), but surgical resection remains the only long-term curative technique. Low grade cases in pediatrics have shown no benefit from adjuvant chemotherapy and post-operative radiotherapy (21–25). There has been no progress in patient survival during the last three decades (26), which is linked to insufficient availability of therapeutics.

The hallmark SS chromosome translocation leads to the expression of a fusion protein consisting of the 379th N-terminal a.a. of SS18 to the 79th C-terminal a.a. of the given SSX fusion partner. These fusion proteins are widely considered to be the main driver of SS pathogenesis (27, 28), as their expression is sufficient to induce SS tumors in mice (29) and their silencing causes SS cells to revert to mesenchymal stem cell-like cells (30). However, neither fusion partner nor the fusion protein possess DNA binding domains (2, 3, 31). Regulation of gene expression by the fusion protein has been determined as indirect via interactions with protein complexes.

Recent evidence supporting epigenetic modifications driving sarcomagenesis (32) and the SS fusion acting as an epigenetic modifier (33, 34) has brought epigenetics to the forefront of the field for SS. Recent efforts have focused on unraveling the mechanism behind the SS18-SSX-mediated epigenetic rewiring.

In this paper, we summarize the evidence of the interplay between the SS fusion protein and the chromatin remodeling machinery with its associated epigenetic modifiers. We will discuss two key protein complex families, SWItch/Sucrose Non-Fermentable (SWI/SNF) and Polycomb Repressive Complexes (PRC) and how they can be targeted to improve current therapies and alleviate/avoid their harmful side-effects (35–38).

## The SWI/SNF Complexes in SS

The SWI/SNF complexes, originally discovered in budding yeast (39–41), have been shown to be evolutionarily conserved, as seen by the presence of homologs of the complex components in *Drosophila* (42) and mammals (43–47). These complexes belong to the Trithorax Group (TrxG) proteins, and utilize ATP hydrolysis to regulate the chromatin state and control transcription (48–50). The SWI/SNF complexes have a canonically activating role, antagonistic to the one of the PRCs, whose description and link to SS is reviewed later.

There are two main mammalian SWI/SNF (mSWI/SNF) complex populations, BAF (BRG1 or BRM associated factors) and PBAF (Polybromo-associated BAF) (49, 51–54). The two populations contain either BRG1 or BRM, which are responsible for the catalytic ATPase activity of the complex, in addition to other core subunits (BAF155, BAF170, BAF47, BAF57) (55). The main difference between the two complexes is the inclusion of BAF250A/B or BAF200, in the BAF and PBAF complexes, respectively (56, 57). Moreover, only BAF complexes contain SS18 and have been shown to interact with the SS fusion proteins. The rest of the 12–14 complex subunits are tissue- and cell-type specific.

Historically, the first evidence for a link between the mSWI/SNF complexes and cancer was shown in Malignant Rhabdoid Tumors (MRT), with the hallmark biallelic inactivation of SMARCB1, the gene coding for the BAF47 protein, observed in 98% of tumors (58–60). This leads to a loss of function of the complexes thus promoting tumourigenesis. Later it was shown that up to 20% of all cancers show mutations in one of the subunits of the mSWI/SNF complexes, making it an extremely disrupted complex, with a key role in cancer (61–64). However, in SS the disruption of the BAF complex does not rely on a loss of function, but on a gain of function. The SS18-SSX fusion proteins have been shown to competitively replace the wild-type SS18 in the BAF complex and excluding BAF47 from it, leading to its proteasomal degradation (7, 65).

These oncogenic BAF complexes are subsequently retargeted to PRC repressed domains and have been shown to activate them. This is evidenced by the presence of BAF complexes on the SOX2 locus, and the decrease in PRC2 deposited tri-methylated lysine 27 on histone 3 (H3K27me3) marks on the same locus (65). This has been confirmed by McBride et al., showing a broad relocalization of the oncogenic BAF complexes in SS, specifically targeting PRC2 repressed domains and recruiting RNA Polymerase II to initiate transcription (66). Furthermore, the H3K27me3 levels increased at PRC2 repressed loci upon SS18-SSX knockdown, reverting back to a non-activated bivalent state (66), characterized by the presence of both activating H3K4me3 and repressive H3K27me3 marks (67). McBride et al. also described a targeting of the PBAF complexes to SS18-SSX activated genes (66), despite the complex not containing SS18, thus not incorporating the fusion protein. This suggests a downstream recruitment of PBAF complex following BAF relocalization.

## The Role of the Polycomb Complexes in SS

**Figure 1:**
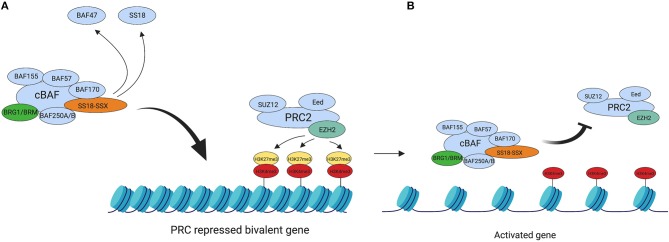
BAF complex retargeting in SS. **(A)** The oncogenic BAF complex, including one of the SS18-SSX fusion proteins is retargeted to PRC2 repressed domains. SS18-SSX inclusion in the BAF complex leads to the eviction of both SS18 and BAF47 from the complex. PRC2 mediates its transcriptional silencing activity via the catalytic subunit EZH2. EZH2 catalyses the tri-methylation of H3K27, which leads to chromatin compaction. **(B)** The oncogenic BAF complex evicts the PRC2 complex, thus repressing its activity and activating the previously bivalent promoter. This leads to an abnormal gene expression profile across the genome due to a widespread epigenetic rewiring and drives tumourigenesis. Created with BioRender.com.

The Polycomb Repressive Complexes 1 and 2 (PRC1 and PRC2) are members of a family of Polycomb group-proteins (PcG proteins). These multiprotein complexes are responsible for chromatin silencing of HOX genes (68, 69) through chromatin compaction (70, 71), mediated by their respective catalytic subunits. The current theory is that the SS18-SSX oncoprotein mediates its transcriptional silencing via interaction with PRC1 and PRC2, since studies have shown SS18-SSX to co-localize with the complexes (33, 72, 73). Figure 1 summarizes the important interplay between PRC2 and BAF for silencing and remodeling of chromatin, respectively.

PRC1 consists of two core subunits: RING1A/B and PCGF1-6. The canonical PRC1 (cPRC1), otherwise known as PRC1.2 or PRC1.4, contains the core subunits RING1A/B, with either polycomb group RING finger protein 2 or 4 (PCGF2 or PCGF4), respectively. These subunits are accompanied by Chromobox domains (Cbx) 2, 4, 6–8 and polyhomeotic homolog proteins 1–3 (HPH1-3) (74–77). There are also several heterogeneous non-canonical PRC1 complexes, however for the purpose of this review, we will be focussing mainly on cPRC1. The function of cPRC1 is the mono-ubiquitination of histone 2A at lysine 119 (H2AK119ub), controlled by the enzyme unit dRing that catalyses the E3 ligase activity (78, 79). This process also involves the Cbx proteins, which are thought to be the determinants for chromatin binding, since they interact with RING1A/B, to form a heterodimer with the core of the cPRC1 complex (74–76). The Cbx chromodomains have been found to preferably localize to H3K27me3 domains (77), which fits with the current hypothesis that PRC1 binds to trimethylated H3K27, in order to maintain suppression of transcription (69, 80–82).

The PCGF components are important for maintaining the protein-protein interactions that initiate chromatin silencing (83) and the knockdown of PCGF4 or either of the RING proteins, leads to a global reduction in H2AK119ub (69). Concurrent with this, Barco et al. also found that SS18-SSX2 influences PCGF4 by interacting with the polycomb complexes to downregulate PCGF4 and subsequently decrease the levels of H2AK119ub (84). This could indicate a method of reprieve from transcriptional silencing by PRC1, utilized by the modified BAF complex and further supports the idea that there is a sophisticated interplay between the different multiprotein complexes in SS.

The previous model suggested that PRC1 and PRC2 complemented each other when repressing chromatin, since many studies have identified that these PcG complexes commonly co-occupy many PcG target loci in *Drosophila* and mice (85–87). The theory was that PRC2 tri-methylated H3K27 and PRC1 maintained this state, due to the binding of a Cbx protein to H3K27me3 and the subsequent PRC1 binding (68, 88–90). However, compelling evidence shows that there may be PRC2 independent pathways for PRC1 chromatin silencing (91).

## The Structure of PRC2 and Its Involvement in SS

PRC2 is another member of the PcG protein complexes. It exerts its chromatin silencing functions via its catalytic subunit Enhancer of Zeste 2 (EZH2) (92, 93), a histone methyltransferase. EZH2 catalyses di- or tri-methylation of lysine 27 on histone 3 (H3K27), to form H3K27me2 or H3K27me3, respectively. SS18-SSX and EZH2 mediate formation of H3K27me3, which leads to repression of tumor suppressor genes such as Early Growth Response 1 (EGR1) (94). The core PRC2 subunits of canonical PRC2 (cPRC2) are EZH2, SUZ12 and Eed (68, 90–95). There are also several PRC2 associated proteins (96, 97) but we will mainly focus on the core components of PRC2, since they are independently sufficient to perform di- and tri-methylation of H3K27 (98).

The previous hypothesis for the pathogenesis of SS was that the SS18-SSX oncoprotein completely knocked out the wild type BAF complex, which then disrupted the balance between the TrxG and PcG proteins (65, 99). It was thought that PRC2 activity was completely unopposed and therefore, EZH2 was able to catalyse over-production of the H3K27me3 motif. This chromatin repression also silences anti-tumourigenic proteins and EZH2 is able to maintain cells in a pluripotent state (100), which would fit with the neural stem-cell like landscape of SS (101, 102). Other cancers display an oncogenic dependency on the PcG complexes (55, 103), however McBride et al. have shown that EZH2 mediated activity may not be the driving factor for the pathogenesis of SS (66). There is currently no definitive theory for the mechanism of SS that accounts for the complex interplay between the TrxG and PcG complexes, therefore a better understanding of the effects and consequences of the expression of SS18-SSX fusion proteins is needed.

## Novel Epigenetic Therapeutic Approaches

**Figure 2:**
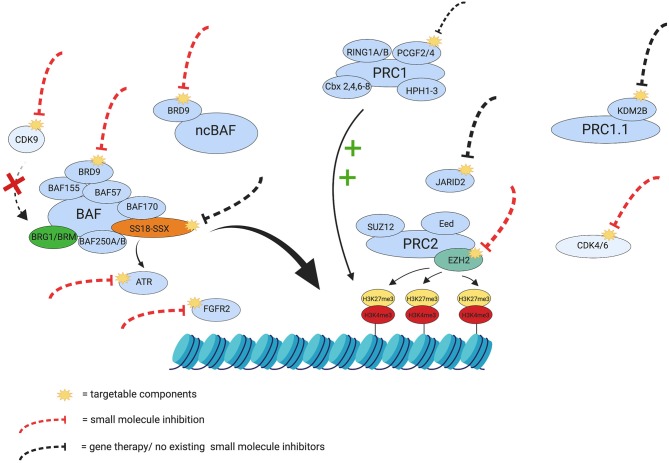
Therapeutic targets in Synovial Sarcoma. The yellow visuals represent the potentially targetable components discussed in this review. The red dashed lines indicate components in SS that already have small molecule inhibitors and the black dashed lines indicate potential targets that currently have no small molecule inhibitor or gene therapy. Certain targets in the figure (e.g., ATR and FGFR2) have been inhibited in other cancers and have shown potential synthetic lethality and growth reduction in SS, respectively, and have therefore been included for consideration as targets. The green plus signs indicate the maintenance of the repressive tri-methylation of H3K27, exerted by PRC1, and the red cross depicts the inhibition of BRG1 through dephosphorylation, should there be inhibition of CDK9. The figure also represents some of the interplay in SS that controls chromatin remodeling and silencing and emphasizes the need for a multifactorial approach to treatment and overcome potential treatment resistance. Created with BioRender.com.

Due to the complexity of SS, its treatment will require multiple combined epigenetic therapies or combinations of targeted therapies, since single therapies have been trialed in the past and have only shown partial success (99, 104). A summary of potential therapeutic targets can be seen in Figure 2.

SS have been shown to have high levels of EZH2 expression and subsequently high H3K27me3 motif expression (92, 99, 105). Moreover, EZH2 expression has been shown to correlate with poor prognosis in these tumors (105). Cho et al. also reported that in sarcomas, an increased expression of PRC2 and it's components were poor prognostic factors for overall survival (106). EZH2 catalyses the di- or tri-methylation of H3K27 via S-Adenosyl Methionine (SAM) and EZH2 expression was also found to be more commonly linked to metastatic SS (8, 106), which emphasizes the importance of EZH2 in metastatic SS. In hypomethylated prostate cancer, the addition of SAM showed a decrease in the proliferative and malignant potential of the cancer cells (107). This highlights the importance of SAM in cancer pathogenesis, therefore trialing a knockdown of SAM in SS should be considered for a combination therapy. Preclinical studies involving EZH2 inhibitors have shown some reduction in cell proliferation in SS cell lines (99, 104), which has prompted investigation in the clinical setting. Indeed, both pediatric and adult patients with refractory SS have been enrolled in phase I and II clinical trials testing the novel EZH2 inhibitor, tazemetostat (ClinicalTrials.gov Identifiers: NCT02601937, NCT02601950, and NCT02875548). Unfortunately, poor initial results in the adult setting were observed (108), which could be explained by the low levels of BAF47 present in SS (65, 109–111). This is further supported by the synthetic lethal effect exerted by combining BAF47 and PRC2 activity repression through EZH2 knockdown or inhibition with another EZH2 inhibitor (EPZ005687) (99). This result is coherent to what has previously been done in MRT with tazemetostat (103). BAF47 is an essential component of the complex under normal conditions (112, 113), but appears redundant for the activity of oncogenic BAF in SS. Instead, the complex relies on its gain of function through the SS18-SSX mediated specific retargeting (66), which explains the poor results shown in EZH2 inhibitor clinical trials.

JARID2 is a PRC2 associated protein (96, 97), which has DNA binding capabilities (114, 115) and has been shown to regulate the trimethyl mark of H3K27 in rhabdomyosarcomas in conjunction with PRC2 (116). Knockdown of JARID2 is linked to a global reduction of PRC2 binding to its target genes (117, 118), which indicates an interdependence for recruitment to target loci. An association between JARID2 and Eed suggests that Eed is the facilitator for the interaction between PRC2 and JARID2 (118), since knockdown of Eed *in vitro* and in *Drosophila* leads to global reduction in H3K27me3 (119). With a potential dependence on Eed, JARID2 has been shown to alter the methylation status of H3K27, thus implicating both Eed and JARID2 as potential targets.

SS differs from rhabdoid tumors and other BAF47 deficient tumors, since its key mechanism is the retargeting of the oncogenic BAF complexes through the 79th C-terminal a.a. of the SSX protein. This is supported by the fact that truncated SSX sequences disrupt the localization of the fusion proteins and inhibit SS proliferation (120), thus Figure 2 shows SS18-SSX to be a target for consideration.

Furthermore, the inhibition of a recently described non canonical BAF (ncBAF) complex (121), distinct from the two previously mentioned BAF and PBAF complexes, has been shown to be a potential synthetic lethal target for SS. The synthetic lethal approach relies on exploiting the vulnerability caused by the inclusion of the fusion genes in the cBAF complex, which becomes unable to perform its canonical role in the cells leading to an increased sensitivity to ncBAF inhibition. The depletion of the BRD9 subunit of the BAF and ncBAF complexes leads to cell death in both SS and MRT (122, 123). This is particularly interesting given the recent development of potent and specific BRD9 targeting compounds (124–128). Another synthetic lethal target for SS is the DNA damage response kinase ATR, which has been shown to impair growth of patient-derived SS xenografts (129). This study has indicated that SS has a dependence on ATR, which is apparent in other cancers and has been trialed in squamous non small cell lung cancer, small cell lung cancer, breast cancer and ovarian cancer (129, 130).

Another potential therapeutic target associated with the BAF complex, is cyclin-dependent kinase 9 (CDK9). It has recently been shown that its inhibition leads to the dephosphorylation of BRG1, one of the catalytic subunits of the BAF complex (131), which impairs its activity and activates silenced genes. The mechanism behind this is not completely understood but given the oncogenic relationship between the BAF complex and the SS fusion, this leads us to believe that it would be worth investigating the effect of CDK9 inhibition in a SS context.

Interestingly, another set of CDKs, namely CDK4 and CDK6, have been shown to be valuable therapeutic targets in SS as well. Targeting the cyclin D1-CDK4/6-Rb axis with the CDK4/6 inhibitor palbociclib leads to proliferation arrest and cell death in SS cell lines (132, 133). Palbociclib was approved for breast cancer treatment by the FDA in 2015 and is currently being trialed in various other cancers (134).

KDM2B is a histone demethylase and is a member of non-canonical PRC1, named PRC1.1 (77, 135). KDM2B is involved in the maintenance of embryonic stem cell (ESC) state in mouse cells (136) and is also implicated in multiple malignancies, such as gynecological, hematological, gastric, and pancreatic cancers (137–139). Its role in these cancers is linked to tumourigenesis and proliferation of the malignant cells, however more recently, KDM2B has been indicated for the maintenance of SS cell transformation (140).

SS18-SSX has also been shown to activate factors that influence lineage, most notably the FGF receptor gene (FGFR2) (101), which is important for inducing a neuronal lineage in stem cells (141). The effector proteins that are influenced by SS could provide another avenue for potential therapeutics. Another consideration for targeting is the protein-protein interaction that the PCGF proteins are responsible for maintaining, since this enables PRC1 mediated chromatin silencing and has not received much consideration previously. More insight into the different subtypes of SS could provide more targets or unknown synthetic lethal targets, however this would require more research into the genetic mechanisms involved in SS.

## Conclusion

The growing evidence surrounding genetic abnormalities inducing a gain of function of the mSWI/SNF complexes, as opposed to the historically identified loss of function in MRT, makes a strong case for further investigation of the mechanism underlying the retargeting of the BAF complex. The interaction between the oncogenic BAF complex and the PcG complexes in SS is not fully understood either and this information may lead to the identification of novel therapeutic targets. There are multiple epigenetic targets discussed in this mini-review that, if targeted in combination, could provide a well-tolerated and safe therapy for pediatric and adult SS. However, a comprehensive understanding of the SS18-SSX induced epigenetic rewiring in SS is needed to allow the most influential epigenetic modifiers to be identified and incorporated into an effective therapy.

## Author Contributions

RH and SS wrote the manuscript. All authors contributed to the manuscript and were involved in revisions and proof-reading. All authors approved the submitted version.

### Conflict of Interest

The authors declare that the research was conducted in the absence of any commercial or financial relationships that could be construed as a potential conflict of interest.
